# Multifunctional behaviors in meta-magnetic shape-memory microwires

**DOI:** 10.1107/S2052252519010856

**Published:** 2019-08-29

**Authors:** Zongbin Li, Claude Esling

**Affiliations:** a Key Laboratory for Anisotropy and Texture of Materials (Ministry of Education), School of Material Science and Engineering, Northeastern University, Shenyang, 110819, People’s Republic of China; b Université de Lorraine, CNRS, Arts et Métiers ParisTech, LEM3, Metz, 57000, France; c Laboratory of Excellence on Design of Alloy Metals for low-mAss Structures (DAMAS), Université de Lorraine, Metz, 57073, France

**Keywords:** meta-magnetic shape-memory microwires, magnetostructural transformation, superelasticity, magnetocaloric effect

## Abstract

Oligocrystalline-structured Ni_43.7_Cu_1.5_Co_5.1_Mn_36.7_In_13_ microwires with bamboo-like grains were successfully prepared by Chen *et al.* [(2019), *IUCrJ*, **6**, 843–853]. Pronounced mechanical and magnetic properties were shown in the tensile superelasticity and the magnetocaloric effect, respectively.

Heusler-type Ni–Mn–*X* (*X* = In, Sn, Sb) alloys are known for their multifunctionality in relation to the martensitic transformation (Kainuma *et al.*, 2006 ▸; Liu *et al.*, 2012 ▸; Sutou *et al.*, 2004 ▸), but their potential applications are limited by their intrinsic brittleness. A promising option is to employ oligocrystalline-structured microwires with bamboo-like grains, because strain incompatibility during deformation and martensitic transformation can be greatly reduced (Ueland *et al.*, 2012 ▸). In this issue of **IUCrJ**, Chen *et al.* (2019 ▸) successfully prepared an Ni_43.7_Cu_1.5_Co_5.1_Mn_36.7_In_13_ microwire using the Taylor–Ulitovsky method (Chiriac & Óvári, 1996 ▸), with the integration of pronounced mechanical and magnetic properties demonstrated by the tensile superelasticity and the magnetocaloric effect, respectively. The realization of multifunctionality in microwires provides a solid foundation for exploiting potential intelligent applications in miniaturized devices under multifield coupling.

Shape-memory alloys represent a unique class of intelligent materials that exhibit shape recovery, as a result of reversible martensitic transformation. Although the large recoverable strains can be obtained easily, a major inconvenience for such shape-memory effects is the low working frequency inherent in thermal control over martensitic transformations. In recent years, a significant breakthrough in the search for high-performance shape-memory alloys is the realization of magnetically driven shape-memory effects (Kainuma *et al.*, 2006 ▸; Ullakko *et al.*, 1996 ▸), enabling large recovery strains and high working frequencies to be obtained simultaneously.

Among the so-called magnetic shape-memory alloys, Heusler-type Ni–Mn–*X* (*X* = In, Sn, Sb) alloys have attracted considerable attention (Kainuma *et al.*, 2006 ▸; Li *et al.*, 2019 ▸; Liu *et al.*, 2012 ▸; Sutou *et al.*, 2004 ▸). A distinct feature of these alloys is that the martensitic transformation involves not only the structural transformation but also the magnetization variation, *i.e.* a magnetostructural transformation from a ferromagnetic austenite to a weak magnetic (antiferromagnetic or paramagnetic) martensite. Such strong coupling between structural order and magnetic order enables the inverse martensitic transformation to be driven by the magnetic field, thus giving rise to the shape-memory effect. Since the magnetic-field-induced magnetostructural transformation is accompanied by meta-magnetic behavior, these alloys are usually termed meta-magnetic shape-memory alloys (Kainuma *et al.*, 2006 ▸). Fig. 1 ▸ illustrates the meta-magnetic transition in the form of isothermal magnetization curves on increasing and decreasing the field. Initially, the magnetic field is applied to the martensite. When the magnetic field reaches a certain critical field μ_0_ *H*_cr_, a sudden change in the slope with progressive increase in magnetization can be observed, indicating the transition from weak magnetic martensite to ferromagnetic austenite. It is noted that Fig. 1 ▸ demonstrates the reversible field-induced magnetostructural transition. On demagnetization, the austenite fully transforms back to the martensite. Associated with such field-induced phase transformations, some other fascinating functional behaviors can also be expected, *e.g.* the magnetocaloric effect, magnetoresistance.

Since Ni–Mn–*X* alloys are typical intermetallic compounds, their poor mechanical properties in polycrystalline alloys, resulting from intrinsic brittleness caused by intergranular fracture (Wang *et al.*, 2006 ▸), strongly constrain their use in a service environment. This dilemma could be mitigated by reducing the sample dimension or sample size, *e.g.* films, wires, particles. When the grain size is comparable to the characteristic sample sizes (*e.g.* film thickness, wire diameter, particle radius) (Dunand & Müllner, 2011 ▸), the strain incompatibilities in the grains are expected to relax at the free surfaces. In this condition, the ratio of surface area to grain boundary area is higher than 1, *i.e.* the so-called oligocrystalline structure. Moreover, such low-dimension or small-sized samples are also quite suitable for miniaturized devices.

In the recent work of Chen *et al.* (2019 ▸), oligocrystalline-structured Ni_43.7_Cu_1.5_Co_5.1_Mn_36.7_In_13_ microwires with a diameter of 100~200 µm were successfully fabricated using the Taylor–Ulitovsky method (Chiriac & Óvári, 1996 ▸), a cost-effective way involving quenching and drawing to prepare the microwires. In the microwires, Co and Cu were added to Ni–Mn–In in order to improve the ferromagnetism of austenite and ductility, respectively. Since the martensitic transformation of microwires occurs around 200 K, the martensitic transformation can be induced by stress at room temperature. As a result, huge tensile superelastic strain up to 13% is achieved in the microwires, in contrast to the poor tensile deformability of bulk alloys. This is because the oligocrystalline structure in the microwires allows the strain accumulated during deformation and transformation to be relieved easily at the free surfaces. Moreover, the structural transformation in the microwires can also be driven by the magnetic field, because of the large magnetization difference between ferromagnetic austenite and weak magnetic martensite, *i.e.* ~90 A m^2^ kg^−1^. In particular, in the temperature range from 160 to 180 K, the reversible field-induced magnetostructural transformation can be induced by a magnetic field of 5 T. Based on this, Chen *et al.* (2019 ▸) also demonstrated a large reversible inverse magnetocaloric effect in the microwires, where a large reversible isothermal entropy change Δ*S*_M_ of 12.7 J kg^−1^ K^–1^ under a field change of 5 T was obtained.

The great improvement of mechanical properties in Ni–Mn–*X* meta-magnetic shape-memory microwires achieved by Chen *et al.* (2019 ▸) represents a significant advance towards potential small scale applications in actuators, sensors and magnetic refrigeration. Above all, the combination of stress and magnetic-field-induced magnetostructural transformation in microwires could provide the ideal prototype materials to simultaneously achieve various caloric effects by utilizing the transformation latent heat, *e.g.* an elastocaloric effect under uniaxial stress, a magnetocaloric effect under magnetic field, and a multicaloric effect under the coupled uniaxial stress and magnetic field. In this context, further alloy design towards high latent heat, narrow hysteresis, low driving force and long fatigue lifetime is suggested.

## Figures and Tables

**Figure 1:**
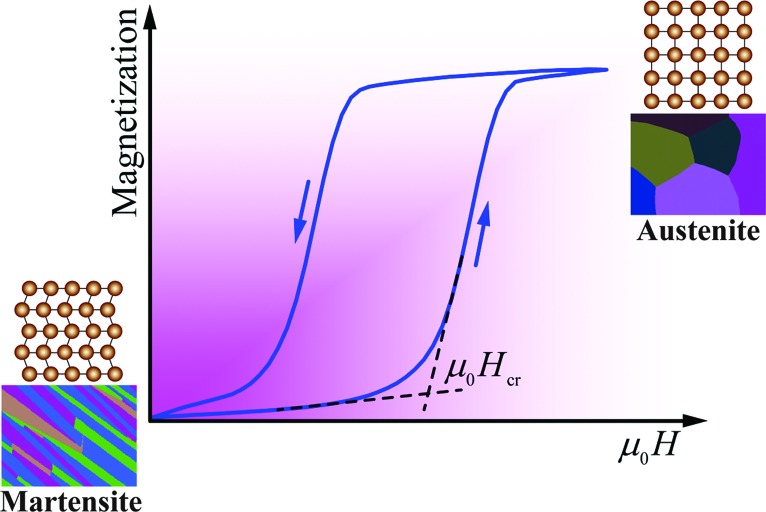
Illustration of field-up and field-down isothermal magnetization curves showing the reversible magnetic-field-induced magnetostructural transformation between weak magnetic martensite and ferromagnetic austenite in Ni–Mn–*X* alloys.
